# Prior water availability modifies the effect of heavy rainfall on dengue transmission: a time series analysis of passive surveillance data from southern China

**DOI:** 10.3389/fpubh.2023.1287678

**Published:** 2023-12-01

**Authors:** Qu Cheng, Qinlong Jing, Philip A. Collender, Jennifer R. Head, Qi Li, Hailan Yu, Zhichao Li, Yang Ju, Tianmu Chen, Peng Wang, Eimear Cleary, Shengjie Lai

**Affiliations:** ^1^Department of Epidemiology and Biostatistics, School of Public Health, Tongji Medical College, Huazhong University of Science and Technology, Wuhan, China; ^2^Department of Infectious Diseases, Guangzhou Center for Disease Control and Prevention, Guangzhou, China; ^3^Division of Environmental Health Sciences, School of Public Health, , University of California, Berkeley, Berkeley, CA, United States; ^4^Division of Epidemiology, School of Public Health, University of California, Berkeley, Berkeley, CA, United States; ^5^Key Laboratory of Land Surface Pattern and Simulation, Institute of Geographic Sciences and Natural Resources Research, Chinese Academy of Sciences, Beijing, China; ^6^School of Architecture and Urban Planning, Nanjing University, Nanjing, China; ^7^State Key Laboratory of Molecular Vaccinology and Molecular Diagnostics, School of Public Health, Xiamen University, Xiamen, China; ^8^WorldPop, School of Geography and Environmental Science, University of Southampton, Southampton, United Kingdom

**Keywords:** dengue, heavy rainfall events, prior water availability, DLNM, Guangzhou

## Abstract

**Introduction:**

Given the rapid geographic spread of dengue and the growing frequency and intensity of heavy rainfall events, it is imperative to understand the relationship between these phenomena in order to propose effective interventions. However, studies exploring the association between heavy rainfall and dengue infection risk have reached conflicting conclusions, potentially due to the neglect of prior water availability in mosquito breeding sites as an effect modifier.

**Methods:**

In this study, we addressed this research gap by considering the impact of prior water availability for the first time. We measured prior water availability as the cumulative precipitation over the preceding 8 weeks and utilized a distributed lag non-linear model stratified by the level of prior water availability to examine the association between dengue infection risk and heavy rainfall in Guangzhou, a dengue transmission hotspot in southern China.

**Results:**

Our findings suggest that the effects of heavy rainfall are likely to be modified by prior water availability. A 24–55 day lagged impact of heavy rainfall was associated with an increase in dengue risk when prior water availability was low, with the greatest incidence rate ratio (IRR) of 1.37 [95% credible interval (CI): 1.02–1.83] occurring at a lag of 27 days. In contrast, a heavy rainfall lag of 7–121 days decreased dengue risk when prior water availability was high, with the lowest IRR of 0.59 (95% CI: 0.43–0.79), occurring at a lag of 45 days.

**Discussion:**

These findings may help to reconcile the inconsistent conclusions reached by previous studies and improve our understanding of the complex relationship between heavy rainfall and dengue infection risk.

## 1 Background

Dengue is a viral infection caused by the dengue virus and transmitted by *Aedes aegypti* and *Ae. Albopictus* mosquitoes (1). The burden of disease attributed to dengue has increased drastically over the past two decades, with the number of cases reported to World Health Organization increasing from 0.5 million cases in 2000 to 5.2 million in 2019 (2). Moreover, the true dengue disease burden is likely to be substantially higher than the reported. Modeling and seroprevalence studies indicate that about 75% of dengue infections may be missed by the surveillance system once they do not present noticeable symptoms (3, 4).

Although a commercial vaccine is available, its application is limited to those who have had prior dengue infection, since the vaccine may increase the risk of severe dengue among those who have not been previously infected (5). As a result, vector control measures remain the primary means for prevention of dengue outbreaks. Therefore, having accurate knowledge of the environmental determinants of mosquito proliferation and dengue virus transmission is essential for reducing the burden of dengue via the design of appropriate vector control measures.

**Figure 1:**
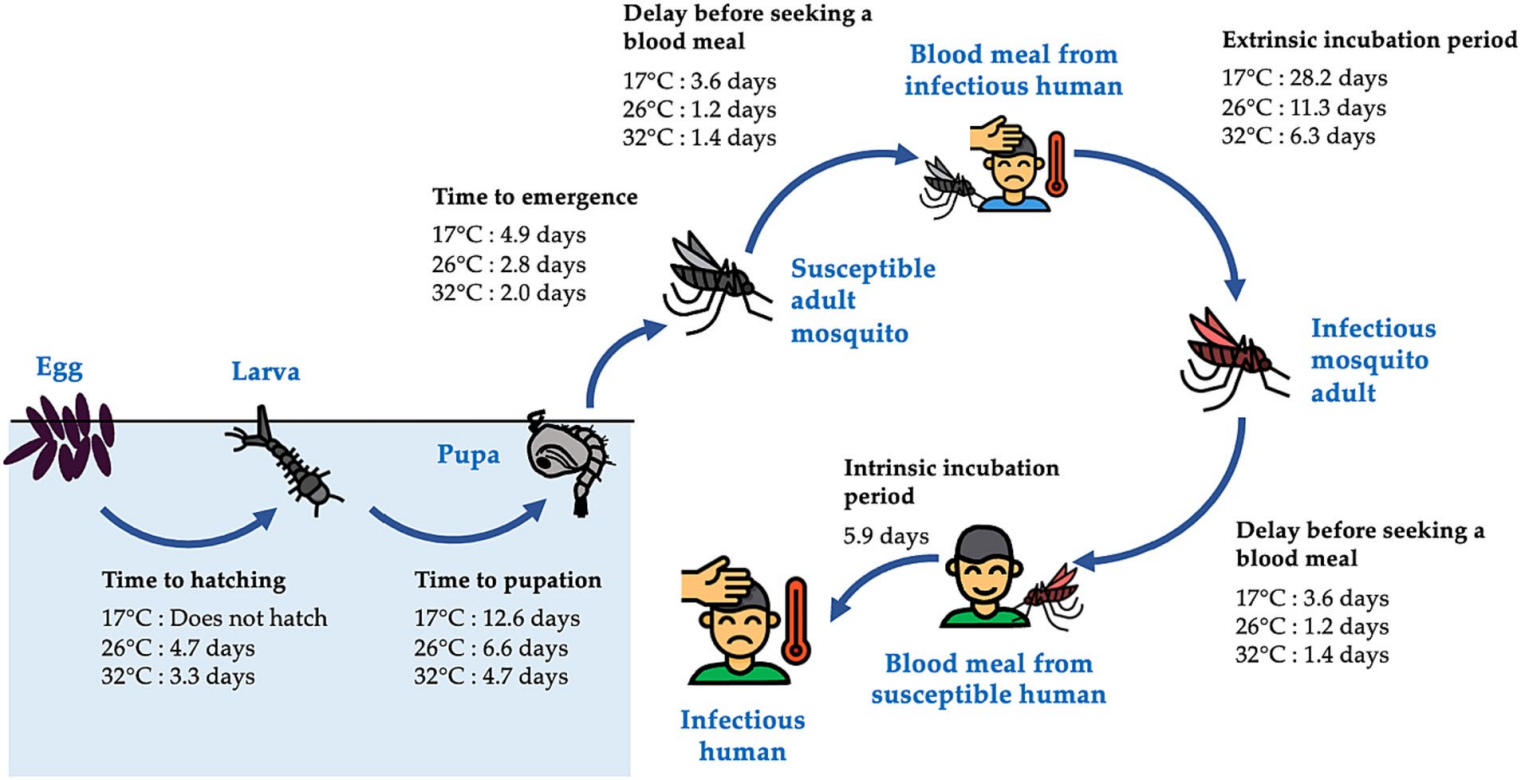
Life stages of the *Aedes* mosquitoes and the transmission of dengue virus. *Aedes* mosquitoes have four distinct life stages, including the aquatic egg, larva, and pupa stages, and the terrestrial adult stage. Upon emergence, female adults seek mates and blood meals before laying eggs. They can become infected with dengue virus by feeding on an infectious human, and, following an incubation period, can transmit the virus to other susceptible humans during subsequent blood meals. Times required for completion of each stage of the cycle at varying temperatures were estimated from enzyme kinetics models fitted to data collected from laboratory studies (6), except for the intrinsic incubation period, which was obtained directly from Chan and Johansson (7). The figure displays times required at 17, 26, and 32°C, which represent the minimum, average, and maximum of daily mean temperatures in Guangzhou, respectively, between August and October, the months during which Guangzhou experiences high dengue case counts (Figure 2C) and frequent heavy rainfall events (Supplementary Figure S2B).

The vectors for dengue, mosquitoes of the genus *Aedes*, undergo four distinct life stages: the aquatic egg, larva and pupa stages, and the terrestrial adult stage (Figure 1). The rate of development between these stages and the mortality rate at each stage depend heavily on temperature and rainfall through complex pathways (8). Higher temperatures can accelerate mosquito development and reproduction, as well as viral replication within mosquitoes, potentially raising the risk of dengue infection (Figure 1). Higher temperatures can also increase mosquito mortality, potentially reducing the risk of transmission (8). Similarly, rainfall has been shown to increase dengue risk by providing additional water for mosquito breeding sites, thereby increasing the environmental capacity for the immature aquatic life stages of mosquitoes (9). On the other hand, it can flush out immature mosquitoes from their breeding sites, thereby impeding the transmission of dengue when the water level in breeding sites is already close to maximum capacity, as demonstrated by field and laboratory experiments (10).

The number of previous studies that have investigated the impact of heavy rainfall events on dengue risk have been limited and have reached inconsistent conclusions. Using data from 35 provinces in Southeast Asia, Wang et al. found that extreme rainfall was associated with a 25 percent reduction in dengue risk (RR: 0.75, 95% CI: 0.62–0.90) compared to no rainfall (11). Conversely, studies in Asia observed a positive association between heavy rainfall and dengue risk (12–15). However, none of the studies mentioned above stratified the analyses by prior water availability, and many were constrained by their reliance on data with low temporal resolution (i.e., weekly or monthly, instead of daily), as well as their limited consideration of the effects of heavy rainfall across multiple time lags.

In this study, we examined the relationship between heavy rainfall and dengue risk using a distributed lag non-linear modeling approach taking into consideration interactions between heavy rainfall events and prior water availability. Using data on the daily number of dengue cases in Guangzhou collected by a passive surveillance system from 2006 to 2018, we aimed to test the hypothesis that prior water availability modified the relationship between heavy rainfall and dengue risk.

## 2 Methods

### 2.1 Study area

**Figure 2:**
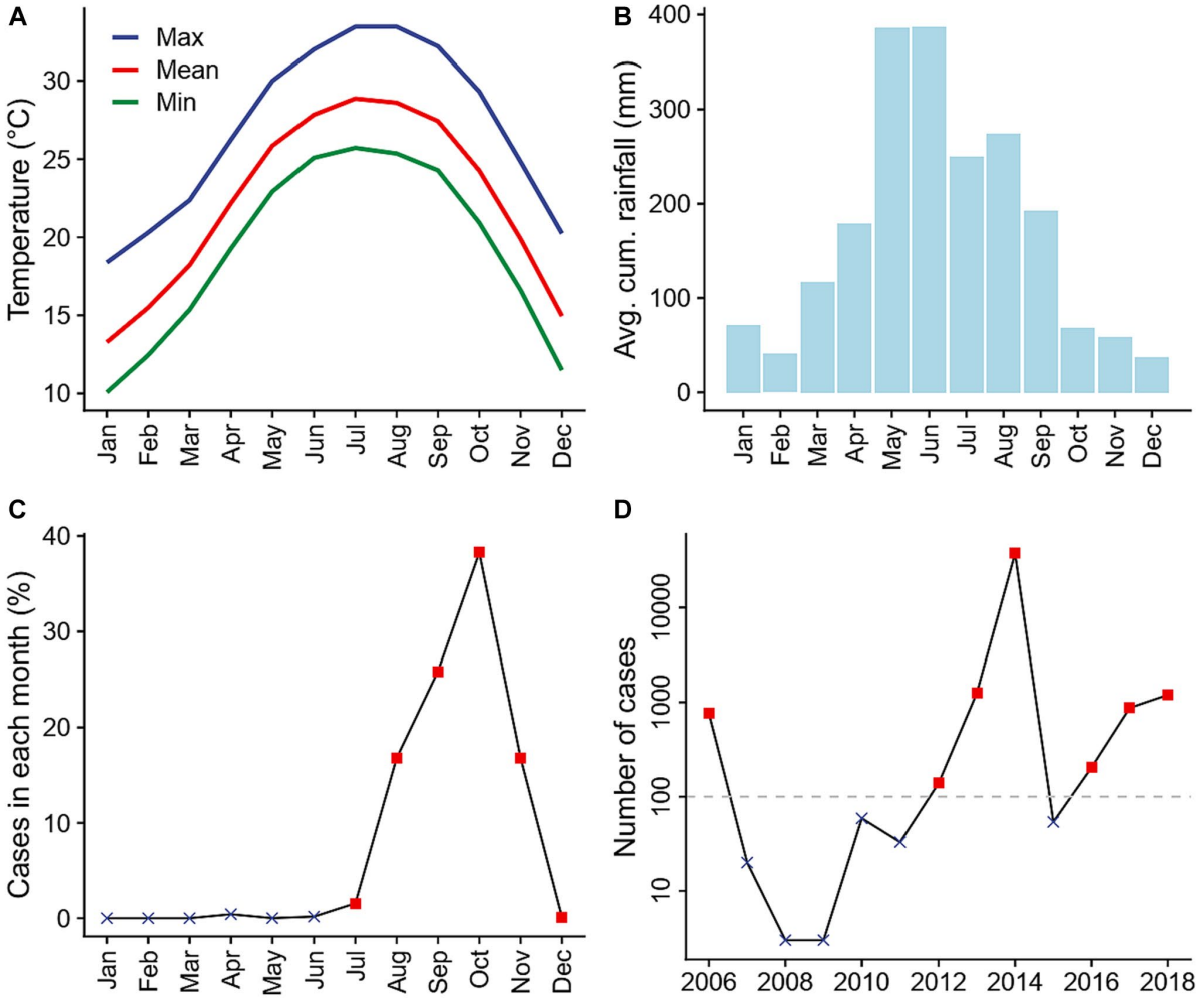
Climate conditions and dengue transmission in Guangzhou prefecture, China between 2006 and 2018. **(A)** Monthly average daily maximum (blue), mean (red), and minimum (green) temperatures. **(B)** Monthly average cumulative rainfall. **(C)** Average percentage of annual dengue cases reported in each month. Red squares represent transmission season months included in the statistical analyses, while black crosses represent those excluded. **(D)** Number of dengue cases reported in each year. Only years with more than 100 reported cases (red squares) were included in the statistical analyses. Climate data was obtained from China Meteorological Data Service Centre, while dengue case count data was obtained from Guangzhou Center for Disease Control and Prevention.

**Figure 3:**
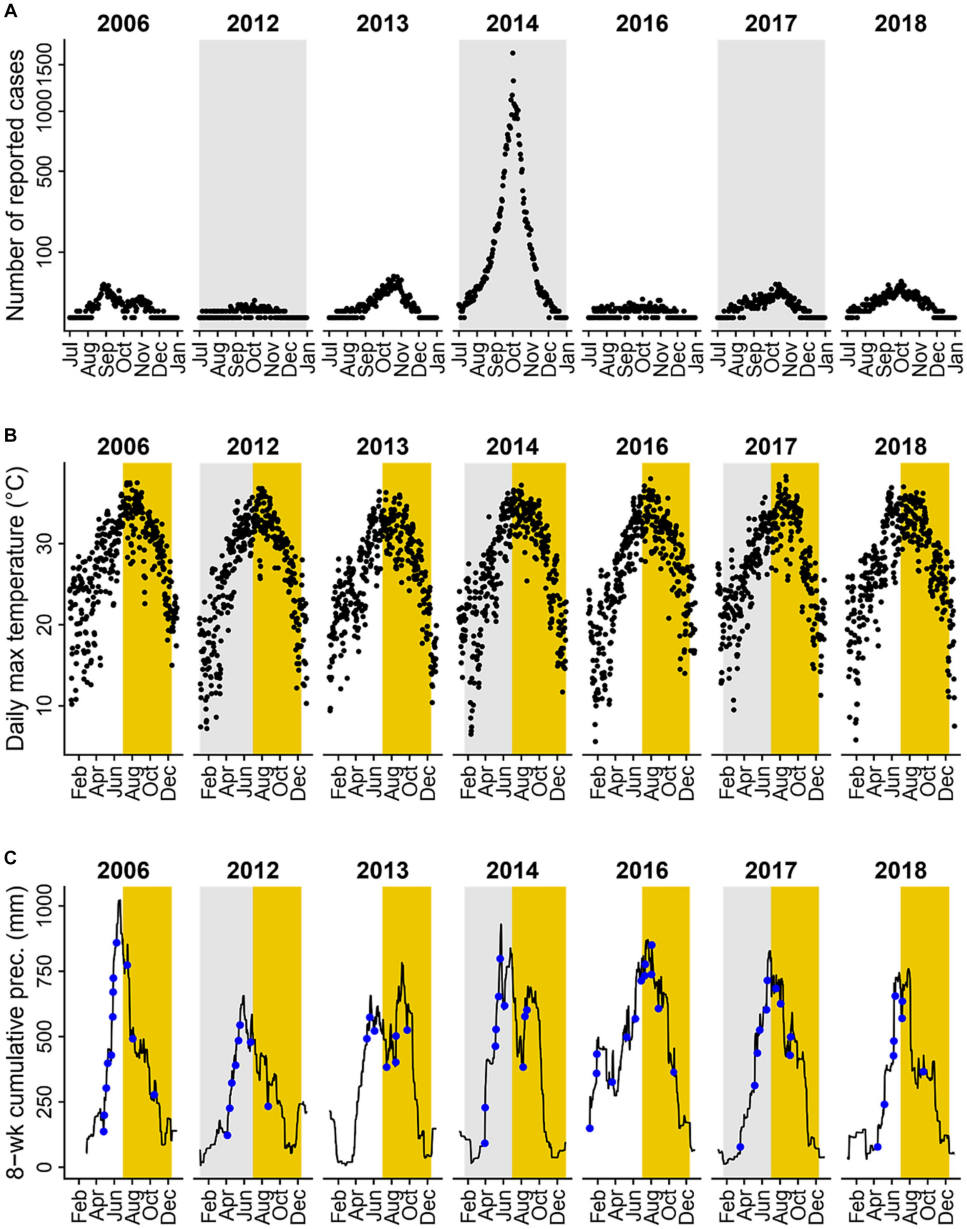
Dengue incidence and climate variables in years where over 100 dengue cases were reported. **(A)** Daily incidence of locally acquired dengue cases reported during transmission seasons (black dots). **(B)** Daily maximum temperature (°C); yellow shaded areas represent the transmission season of July to December. **(C)** Daily prior water availability (defined as 8-week cumulative precipitation; black line) and heavy rainfall events (defined as daily precipitation levels exceeding the 95th percentile value of rainy days in the study period, 51 mm, blue dots); yellow shaded areas represent the transmission seasons of July to December. Dengue incidence and climate variables for all years and months between 2006 and 2018 are shown in Supplementary Figure S1.

Guangzhou, a subtropical prefecture located in southern China, has the fifth largest population (18.7 million) and the fourth highest gross domestic product across all prefectures in the country (16). The burden of dengue in this region is high: between 1990 and 2015, dengue cases in Guangzhou accounted for over 65 percent of dengue cases reported in China (6, 17). Guangzhou is located around the Tropic of Cancer and has a typical subtropical climate with hot and humid summers and mild and dry winters (Figures 2A,B), which is favorable for mosquito growth. Heavy rainfall is common in summer months (Figure 3C; Supplementary Figure S2B). Notably, unlike most other dengue transmission areas, where the primary vector is *Ae. aegypti*, the sole vector in Guangzhou is *Ae. albopictus*, which tolerates cold during the winter months better through egg diapause (18). Dengue transmission in Guangzhou follows a clearly defined seasonal pattern. No local transmission is reported during the winter months, when the climate is cooler, and over 99 percent of cases are reported between July and December, when the climate is more favorable by the vectors (Figure 2C). Each year, the initiation of local transmission requires the arrival of imported cases (17, 19). The starting time of local transmissions play a crucial role in determining the outbreak size of that year, with earlier local transmission associated with larger outbreaks (6). As a result, the number of cases varies significantly from year to year. For example, 37,338 cases were recorded in 2014 (89 percent of the 41,939 cases recorded during the 2006–2018 study period) while only three cases were reported during 2008 and 2009 (Figure 2D).

### 2.2 Data collection and processing

Dengue is a notifiable disease in China. Cases are diagnosed according to the National Diagnostic Criteria for Dengue Fever (WS216-2008) (20). Once diagnosed, cases are required to be reported within 24 h via the web-based National Infectious Disease Reporting System that covers almost all healthcare facilities in China (20, 21). Travel history is a mandatory reporting field, and cases where the patient did not report travel to dengue endemic areas in the 15 days prior to the onset of illness are considered to be locally-acquired (21). We obtained daily counts of locally acquired incidence cases, including both clinically-diagnosed and laboratory-confirmed infections, in Guangzhou throughout the period between January 1st, 2006 to December 31st, 2018 from the Guangzhou Center for Disease Control and Prevention. Year-end population data for Guangzhou from 2005 to 2018 were collected from the Guangdong Statistical Yearbooks (22) and used to interpolate the population size on each day linearly.

Daily minimum, mean, and maximum temperature and cumulative precipitation between 2006 and 2018 from a weather station in Guangzhou were collected from the China Meteorological Data Service Centre.^1^ We defined a heavy rainfall event as a day when total precipitation exceeded the 95th percentile of all non-zero daily precipitation values in the study period (13, 14). The resulting cutoff of 51 mm corresponds well with the definition used by the China Meteorological Administration, which considers a heavy rainfall event as cumulative rainfall exceeding 50 mm within 24 h (23). We used the cumulative precipitation during the preceding 8 weeks as a proxy measure of water availability in mosquito breeding sites (hereafter referred to as prior water availability) following previous literature (24, 25).

### 2.3 Statistical analysis

We constructed a distributed lag non-linear model (DLNM) to examine the effects of temperature, heavy rainfall, prior water availability, and the interaction between heavy rainfall and prior water availability on dengue incidence at multiple lags. We used the distributed lag model to account for the delayed effects of climatic and hydrological factors on dengue infection risk. We assumed the outcome followed a negative binomial distribution to account for potential overdispersion of daily case counts. We specified the DLNM as:


$$Y_{t}\sim \mathrm{NB}\left(\mu_{t},k\right)$$

(1) $$\begin{array}{l} \log\left(\mu_{t}\right)=\alpha_{0}+\mathrm{ns}\left(t\right)+{\mathrm{DOW}}_{t}+\mathrm{cb}\left({\mathrm{Temp}}_{t}\right)+\mathrm{cb}\left({\mathrm{HeavyRain}}_{t}\right)+\\ \mathrm{cb}\left({\mathrm{WaterLevel}}_{t}\right)+\mathrm{cb}\left({\mathrm{HeavyRain}}_{t}*{\mathrm{WaterLevel}}_{t}\right)+\\ \mathrm{offset}\left(\log\left({\mathrm{population}}_{t}\right)\right) \end{array}$$

where NB(•) represents the negative binomial distribution; $Y_{t}$ and $\mu_{t}$ represent the number of observed and expected locally-acquired dengue cases whose symptoms onset on day t, respectively; *k* is the dispersion parameter of the negative binomial distribution; $\alpha_{0}$ is the model intercept; ns(t) is a natural spline smooth function of prospective time with 7 degrees of freedom per year, included to control for long-term and seasonal trends of any unmeasured confounders as in a majority of the existing environmental epidemiology studies (26–28); and DOW (day-of-week) is a categorical variable for day of the week, included to control for the day of week effects in reporting and incidence. The symbol cb(•) denotes the cross-basis function in DLNM, which models the smooth effect surface in both lag and variable value dimensions. In the lag dimension, we modeled the delayed effects of temperature ( ${\mathrm{Temp}}_{t}$ ), heavy rainfall events ( ${\mathrm{HeavyRain}}_{t}$ ), prior water availability ( ${\mathrm{WaterLevel}}_{t}$ ) and the interaction between heavy rainfall events and prior water availability ( ${\mathrm{HeavyRain}}_{t}*{\mathrm{WaterLevel}}_{t}$ ) with natural cubic splines with two internal knots equally placed in the log range of lags (29, 30). We used a maximum lag of 140 days (i.e., 20 weeks) based on the results of previous studies about the lagged effects of climatic factors on dengue transmission (12, 31). In the variable value dimension, we modeled the effects of temperature with a natural cubic spline with two internal knots equally placed along the range of observed temperatures (29, 30), the effect of a heavy rainfall using binary indicators for occurrence of heavy rainfall events, and effects of prior water availability and their modification by heavy rainfall as linear functions (24). To account for shifts over time in the population at risk, a population offset term $\log\left({\mathrm{population}}_{t}\right)$ was included. Daily maximum temperature, rather than the mean or minimum temperature, was used in the model since it exhibited the lowest collinearity with the other variables. We used ns(t) instead of ns(year)+ns(dayof the year) to control for long-term and seasonal trends of any unmeasured confounders, since the seasonal pattern of dengue incidence rates in Guangzhou varied from year to year, and the model with flexible seasonal terms ns(t) had a remarkably lower deviance than the model with a fixed seasonal term ns(year)+ns(dayof the year) (4749.0 vs. 5807.3). To obtain the effects of heavy rainfall events under the *low*, *medium*, and *high* prior water level scenarios, we centered ${\mathrm{WaterLevel}}_{t}$ at its 5th, 50th, and 95th percentile values between 2006 and 2018 (25.0, 260.6, and 786.9 mm) in three separate model fits. In doing so, the cross basis on heavy rainfall events can be interpreted as the delayed effects of a heavy rainfall event when prior water availability is at the centered value (either high, medium, or low), as the interaction term in Equation ((1) will equal zero. We restricted our analysis to local transmission months (i.e., July–December) in years with over 100 reported cases (2006, 2012–14, and 2016–18, Figure 2D) to examine effects of heavy rainfall events and prior water availability on dengue only among time periods with continuous local transmission. Cases during these periods contributed over 99 percent of all cases between 2006 and 2018.

### 2.4 Sensitivity analyses

To test the robustness of our findings, we conducted sensitivity analyses that explored the results with alternative centering values for prior water availability (such as using the 15th, 25th, …, 85th percentile values); definitions of heavy rainfall events (daily precipitation levels above the 90th percentile value on rainy days within the study period, which is 34.6 mm); number of preceding weeks over which to aggregate measures of prior water availability (7 or 9 weeks); degrees of freedom used for controlling long-term and seasonal trends of potential unmeasured confounders (8 or 9 degrees of freedom per year); and numbers of knots for the lag-response relationship of heavy rainfall events (3 or 4 knots). We also examined the results when excluding data in 2014 from the analyses due to its notably high number of reported cases (Figure 2D).

All analyses were conducted in R 4.2.1 (32) with packages tidyverse 1.3.2 (33), data.table 1.14.2 (34), and lubridate 1.8.0 (35) for data processing, tsModel 0.6–1 (36), splines 4.2.1 (37), dlnm 2.4.7 (38) and INLA 22.07.23 (39) for statistical modeling, and ggplot2 3.3.6. (40), ggsci 2.9 (41) and cowplot 1.1.1 (42) for visualization. All data and code are available from https://github.com/qu-cheng/dengue_heavyrain.

## 3 Results

From 2006 to 2018, a total of 41,939 locally acquired and clinically-diagnosed or laboratory-confirmed dengue cases were reported in Guangzhou. The number of newly-onset cases reported varied across years, with 37,338 cases reported in 2014, and only 3 cases reported in both 2008 and 2009. Seven years (2006, 2012–2014, and 2016–2018) met our inclusion criteria of having more than 100 cases reported. Figure 3A illustrates the number of dengue cases reported per day between July and December for included years.

In years with more than 100 reported dengue cases, daily maximum temperature ranged from 5.6 to 38.3°C, with an average of 26.8°C and a median of 27.9°C (Figure 3B). The number of heavy rainfall events varied year-to-year (Figure 3C; Supplementary Figure S2A), with 2016 having the most (13 events) and 2013 having the least (7 events). Heavy rainfall events were most frequent between April and September, and least frequent between November to February (Supplementary Figure S2B), with two seasonal peaks of event frequency in May and August. Average prior water availability, calculated as the cumulative precipitation during the previous 8 weeks, was at its highest in 2016 and its lowest in 2012 among years included in the statistical analyses (Figure 3C; Supplementary Figure S3A). Across months, prior water availability was greatest from May to October and lowest from November to March (Supplementary Figure S3B). Mean 8-week cumulative rainfall exhibited a single seasonal peak in June.

**Figure 4:**
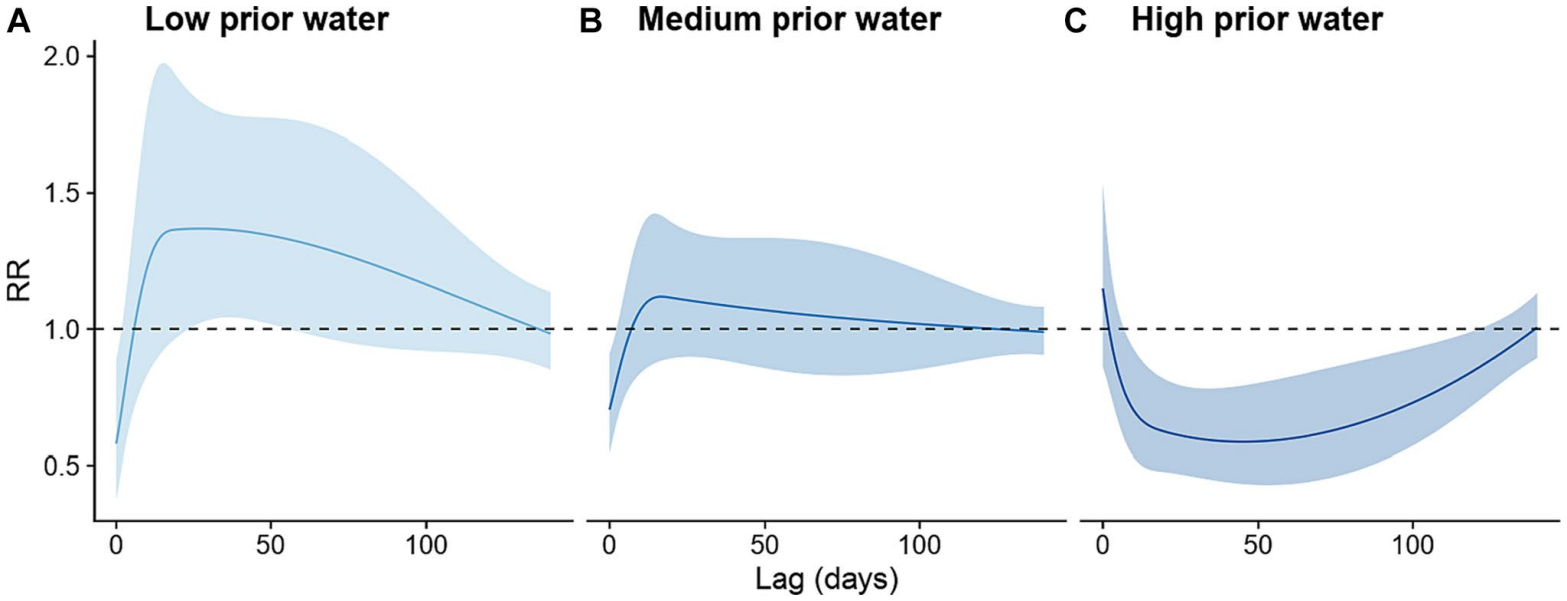
Lagged effects of heavy rainfall events on dengue infection risk stratified by prior water availability scenarios. **(A)** *Low* prior water availability scenario, defined as cumulative rainfall during the preceding 8 weeks at its 5th percentile value; **(B)** *Medium* prior water availability scenario, defined as cumulative rainfall during the preceding 8 weeks at its 50th percentile value; and **(C)** *High* prior water availability scenario, defined as cumulative rainfall during the preceding 8 weeks at its 95th percentile value.

The effects of heavy rainfall on the risk of dengue infection were modified by prior water availability. When the cumulative rainfall during the preceding 8 weeks was at its 5th percentile value (indicating *low* prior water availability), the occurrence of heavy rainfall events was associated with increased risk of dengue infection at a lag of 24–55 days. The strongest increase in dengue incidence occurred 27 days after a heavy rainfall event (incidence rate ratio, IRR: 1.37, 95% CI: 1.02–1.83, Figure 4A). By contrast, when the cumulative rainfall during the preceding 8 weeks was at its 95th percentile value (indicating *high* prior water availability), the occurrence of heavy rainfall events was associated with reduced dengue risk at a lag of 7–121 days, with the strongest negative effect occurring 45 days after the heavy rainfall events (IRR: 0.59, 95% CI: 0.43–0.79, Figure 4C). The shape of the lag-response curve of heavy rainfall events under *medium* prior water availability scenario was very similar to that of the *low* prior water availability scenario. However, the delayed effects of heavy rainfall events observed under *medium* prior water availability on dengue incidence were closer to the null (Figure 4B).

To identify the level of prior water availability at which the effect of heavy rainfall events switches from increasing to decreasing dengue incidence rates, we examined other centering values for the cumulative rainfall in the proceeding 8 weeks (i.e., the 15th, 25th, …, 85th percentile values, Supplementary Figure S5). Using the RR at a 35-day lag, we found that the association between heavy rainfall events was positive when prior rainfall availability was below the 15th percentile, null when prior rainfall availability was between the 25th and 75th percentile, and negative when prior rainfall availability was above the 85th percentile.

**Figure 5:**
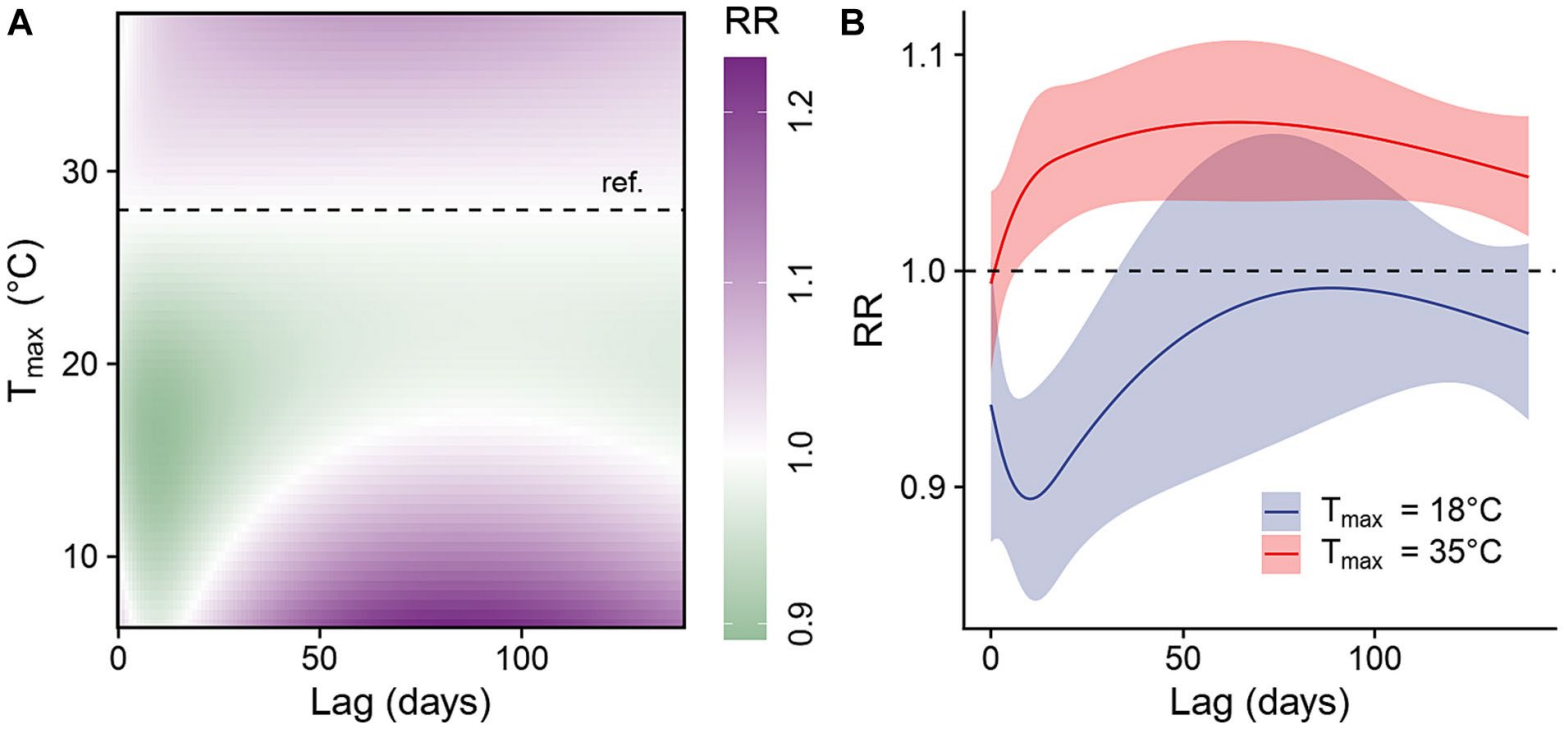
Lagged effects of daily maximum temperature on dengue infection risk. **(A)** Contour plot of the association between daily maximum temperature and the risk of dengue infection at various lag times, relative to the study period’s median daily maximum temperature of 28°C (horizontal dashed line). **(B)** Lag-response association for low (18°C, the 10th percentile of daily maximum temperature observed between 2006 and 2018) and high (35°C, the 90th percentile of daily maximum temperature observed between 2006 and 2018) daily maximum temperature relative to median daily maximum temperature (28°C).

We examined the lag-response relationship for different temperatures compared to 28°C, the median daily maximum temperature observed between 2006 and 2018. Our findings revealed that lower temperatures were associated with a reduced risk of dengue infection, while higher temperatures were associated with an increased risk (Figure 5). When compared with the reference temperature, a daily maximum temperature at 18°C, the 10th percentile of daily maximum temperature observed between 2006 and 2018, was associated with reduced dengue infection risk at lags of 1–32 days. The strongest negative association occurred at a lag of 10 days (IRR: 0.89, 95% CI: 0.85–0.94, Figure 5B). In comparison, a daily temperature of 35°C, the 90th percentile of daily maximum temperature observed between 2006 and 2018, was associated with increased risk of dengue infection at lags of 7–140 days, with the strongest positive association occurring at a lag of 63 days (IRR: 1.07, 95% CI: 1.03–1.11) (Figure 5B).

Varying thresholds used to define heavy rainfall (Supplementary Figure S6), weeks over which prior precipitation was aggregated to estimate water availability (Supplementary Figure S7), degrees of freedom in the temporal trend term (Supplementary Figure S8), knots used to model the lag-response of temperature, heavy rainfall events, and prior water availability (Supplementary Figure S9), and excluding data from 2014 (Supplementary Figure S10) did not qualitatively change the conclusion of our analyses. Across these scenarios, we consistently observed a positive association between heavy rainfall events and dengue infection risk when prior water availability was low, and a negative association when prior water availability was high, although the strength of the associations varied. Comparable to the baseline scenario, the occurrence of a heavy rainfall event was generally associated with a significant change in dengue risk at a lag of 25–45 days. The exact lag days with significant associations, the lag day with the strongest association (including both positive and negative association), and the relative risk and its 95% CI on the lag day with the strongest association are shown in Supplementary Table S1.

## 4 Discussion

As the climate changes, heavy rainfall events are projected to increase in frequency and intensity globally (43). Understanding the effects of heavy rainfall events on dengue transmission is critical for developing targeted public health interventions to reduce the risk of dengue transmission following extreme weather events. Heavy rainfall has long been hypothesized to increase dengue risk when water availability is low by creating more mosquito habitats. However, risk may decrease when water availability is high as immature aquatic stages of mosquitoes are flushed out of existing habitats (44, 45). To the best of our knowledge, this hypothesis had not been formally tested with observational data up to this point. Using daily weather and dengue case data from Guangzhou, China, we developed a distributed lag non-linear model and found evidence supporting this theory. Our results suggest that heavy rainfall events were likely to increase dengue risk at a lag of 25–45 days (3–7 weeks) when water availability before the heavy rainfall was medium to low, but decrease dengue risk when prior water availability was high.

This lag period corresponds well with the time from development of immature aquatic stages to the onset of human dengue cases at 26°C, the average daily mean temperature in Guangzhou between August and October. These months observe high dengue case counts and frequent heavy rainfall events in our study area. According to a previous study using enzyme kinetic models fit to data collected from laboratory studies (6), at 26°C, the times required for the development of eggs into larvae, larvae into pupae, and pupae into adults are 5, 7, and 3 days, respectively; the average time required for *Ae. albopictus* to find a host for a blood meal is 1 day, meaning at least 2 days would be required for a mosquito to acquire the virus from a human and transmit it to another; and the average length of the extrinsic and intrinsic incubation periods are 11 and 6 days (7), respectively (Figure 1). Together these processes take 22 days when counting from the last aquatic stage (pupa), or 34 days when counting from the first aquatic stage (egg). The concordance between these time periods and the lag times identified by our study (25–45 days) suggest that heavy rainfall events can influence various stages of the entire process.

Although some research has shed light on the effects of heavy rainfall on dengue infection risk, results have been inconsistent and heterogenous between study locations. For instance, a study conducted across all 21 prefectures in Guangdong Province, China, found that heavy rainfall events were significantly associated with increased dengue risk in seven prefectures (including Guangzhou) yet reduced dengue risk in four prefectures (13). Another study across 35 provinces in Southeast Asia observed a negative association between heavy rainfall and dengue risk in Sri Lanka, with no significant associations in Malaysia, Thailand, and Singapore (11). Stratifying data by prior water availability could potentially result in more consistent conclusions.

Our findings suggest that warmer daily maximum temperatures are linked to a higher risk of dengue infection, with the delayed effects of warmer temperatures likely to persist for several months. These results align with earlier studies conducted in regions with a subtropical climate. For example, a study in Guangzhou observed the highest dengue risk between 20 and 45 days after a daily maximum temperature of 30 to 35°C, with the lagged effects lasting up to 141 days (46). Similarly, a study in Barbados identified a positive association between monthly minimum temperature and dengue infection risk, with peaks occurring after 1–2 months (29). Our results also make sense in the context of the mosquito and dengue lifecycle, as temperatures in the upper range observed in our study region are hypothesized to enhance nearly all stages, ranging from egg development to virus replication (Figure 1). In contrast, research from tropical regions has suggested an inverted U-shaped relationship between temperature and dengue risk, where relative risk increases first before decreasing (11, 47, 48). While these results suggest that extreme heat may hinder parts of the mosquito or viral lifecycle, the optimal temperature for mosquito development and virus transmission may vary depending on the location (11, 47, 48).

Some limitations should be acknowledged for this study. First, our findings rely on data acquired via passive surveillance, which may be influenced by local healthcare-seeking behavior, disease reporting practices, and the clinical severity of disease. However, these factors seem unlikely to change with the exposure status, meaning that any resulting bias would be expected to pull effect estimates toward the null. Second, because of data availability, our study was limited to a single subtropical prefecture in southern China, which has different seasonal patterns in rainfall compared to other dengue transmission hotspots in tropical regions. While we expect that effect modification by prior water availability of the association between heavy rainfall events and dengue incidence may work similarly in other locations, further research from diverse settings is needed to confirm this and to estimate location-specific effects.

## 5 Conclusion

Our study has shown that the impact of heavy rainfall events on dengue risk relies on prior water availability. To gain a deeper understanding of the modification effect of prior water availability, it is essential to conduct future studies that go beyond the scope of our research. These studies include epidemiological investigations conducted in regions beyond southern China, allowing for a broader assessment of our hypothesis. Additionally, direct observational studies on mosquito density would provide valuable insights into the association between prior water availability and mosquito populations. These findings may help to reconcile the inconsistent conclusions reached by previous studies and improve our understanding of the complex relationship between heavy rainfall and dengue risk. Moreover, they can provide support for the development of more precise prevention strategies during extreme events under future climate conditions.

## Data availability statement

All data and code used is publicly available via https://github.com/qu-cheng/dengue_heavyrain. Further inquiries should be directed to the corresponding author(s).

## Ethics statement

The studies involving humans were approved by Ethics Committee of the Guangzhou Center for Disease Control and Prevention. The studies were conducted in accordance with the local legislation and institutional requirements. The ethics committee/institutional review board waived the requirement of written informed consent for participation from the participants or the participants’ legal guardians/next of kin because Collecting routine infectious disease surveillance data from hospitals.

## Author contributions

QC: Conceptualization, Formal analysis, Funding acquisition, Methodology, Software, Visualization, Writing – original draft, Writing – review & editing. QJ: Conceptualization, Data curation, Resources, Writing – review & editing. PC: Methodology, Visualization, Writing – review & editing. JH: Methodology, Visualization, Writing – review & editing. QL: Writing – review & editing. HY: Writing – review & editing. ZL: Writing – review & editing. YJ: Writing – review & editing. TC: Writing – review & editing. PW: Methodology, Writing – review & editing. EC: Methodology, Writing – review & editing. SL: Methodology, Writing – review & editing.

## Footnotes

^1^ http://data.cma.cn/
