# Supplementary material for: Prior water availability modifies the effect of heavy rainfall on dengue transmission: a time series analysis of passive surveillance data from southern China
Source: Front Public Health. 2023 Dec 1;11:1287678. doi: 10.3389/fpubh.2023.1287678 (PMC10722414; doi:10.3389/fpubh.2023.1287678)
Supplement: Supplementary file 1 [file Data_Sheet_1.pdf]

Supplementary Information for

Prior water availability modifies the effect of heavy rainfall on dengue transmission

Qu Cheng[1#]*, Qinlong Jing[2#], Philip A. Collender[3], Jennifer R. Head[4], Qi Li[1], Hailan Yu[1], Zhichao Li[5], Yang Ju[6], Tianmu Chen[7], Peng Wang[1], Eimear Cleary[8], Shengjie Lai[8]

1 Department of Epidemiology and Biostatistics, School of Public Health, Tongji Medical College, Huazhong University of Science and Technology, Wuhan 430030, China

2 Department of Infectious Diseases, Guangzhou Center for Disease Control and Prevention, Guangzhou 510440, China

3 Division of Environmental Health Sciences, School of Public Health, University of California, Berkeley, Berkeley 94720, United States of America

4 Division of Epidemiology, School of Public Health, University of California, Berkeley, Berkeley 94720, United States of America

5 Key Laboratory of Land Surface Pattern and Simulation, Institute of Geographic Sciences and Natural Resources Research, Chinese Academy of Sciences, Beijing 100101, China

6 School of Architecture and Urban Planning, Nanjing University, Nanjing, 210023, China

7 State Key Laboratory of Molecular Vaccinology and Molecular Diagnostics, School of Public Health, Xiamen University, Xiamen 361102, China

8 WorldPop, School of Geography and Environmental Science, University of Southampton, Southampton, SO17 1BJ, UK

[*] Corresponding author: Qu Cheng, chengqu@hust.edu.cn

[#] Contributed equally to this work

Table of contents

Figure S1. Descriptive results of the disease and climate data for all years between 2006 and 2018

Figure S2. Number of heavy rainfall events across years or months

Figure S3. Mean 8-week cumulative rainfall across years or months

Figure S4. Lagged effects of heavy rainfall events on dengue infection risk by prior water availability scenarios when the centering value for the cumulative rainfall during the preceding 8 weeks changed between its 5th to 95th percentile.

Figure S5. Lagged effects of heavy rainfall events on dengue infection risk by prior water availability scenarios when heavy rainfall was defined as daily precipitation above its 90th percentile value on rainy days in the study period (34.6 mm)

Figure S6. Lagged effects of heavy rainfall events on dengue infection risk by prior water availability scenarios when prior water availability was represented by the cumulative rainfall in the previous 7 or 9 weeks.

Figure S7. Lagged effects of heavy rainfall events on dengue infection risk by prior water availability scenarios when the degrees of freedom per year was set to 8 or 9

Figure S8. Lagged effects of heavy rainfall events on dengue infection risk by prior water availability scenarios when 3 or 4 knots was used to specify the lag-response relationship of heavy rainfall events

Figure S9. Lagged effects of heavy rainfall events on dengue infection risk by prior water availability scenarios when data from the year 2014 was excluded from the analyses

Table S1. Summary results of the sensitivity analyses

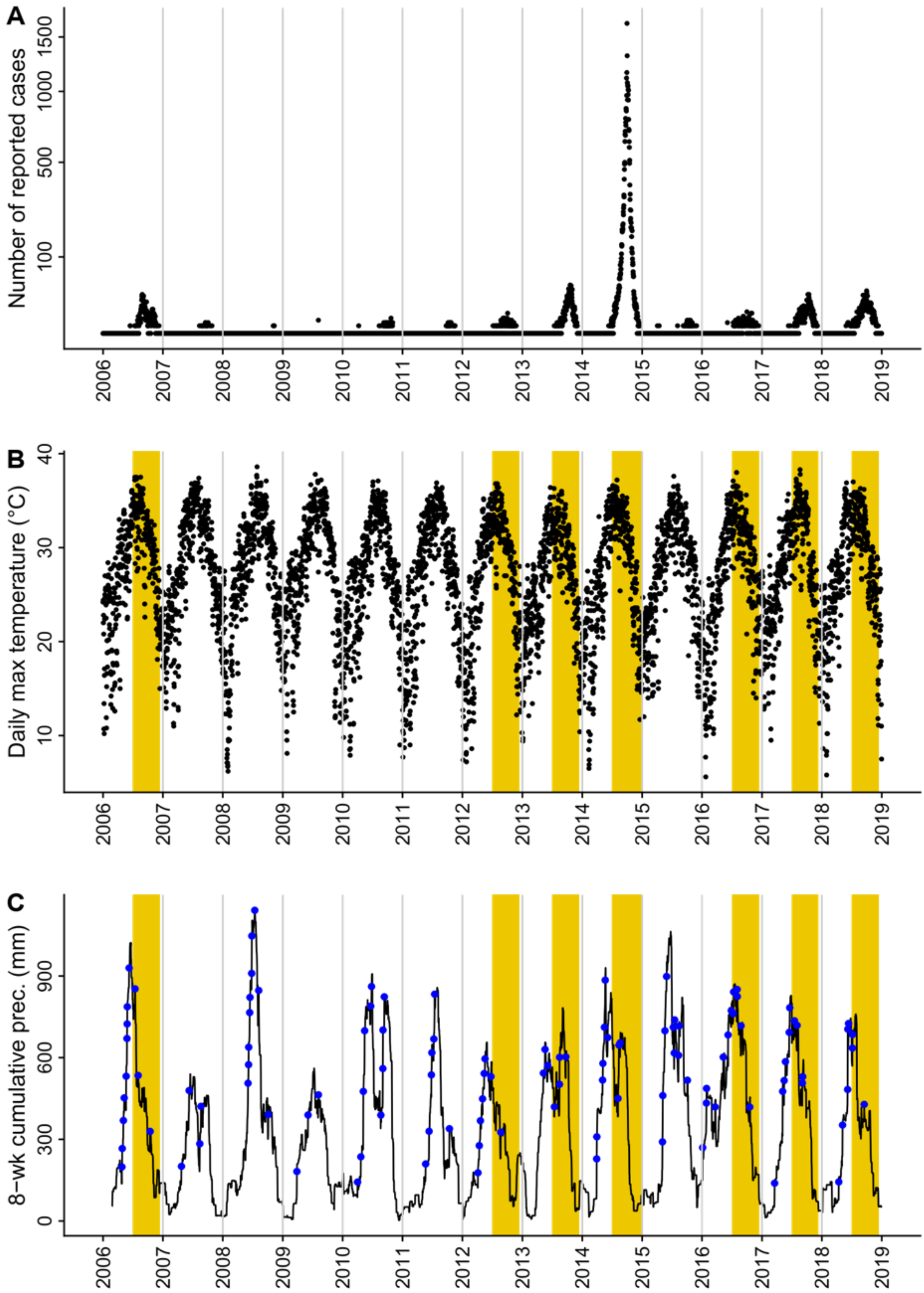

Figure S1. Dengue incidence and climate variables for all years between 2006 and 2018. (A) Daily incidence of locally acquired dengue cases (black dots). (B) Daily maximum temperature (°C); yellow shaded areas represent the transmission season of July to December. (C) Daily prior water availability (defined as 8-week cumulative precipitation; black line) and heavy rainfall events (defined as daily precipitation levels exceeding the 95th percentile value of rainy days in the study period, 51 mm, blue dots); yellow shaded areas represent

the transmission seasons of July to December.

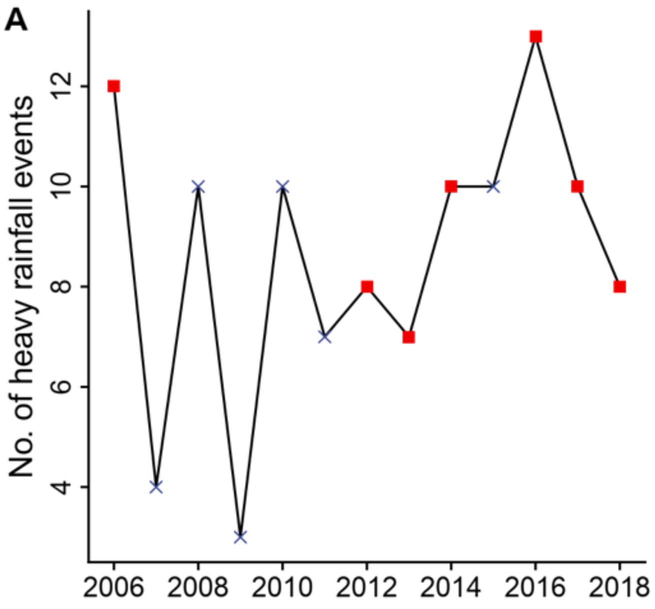

Figure S2. Number of heavy rainfall events across years or months. (A) Total number of heavy rainfall events (defined as having a daily precipitation above its 95th percentile value on rainy days in the study period, 51 mm, blue dots) in each year. Red squares correspond to years with more than 100 cases, which were included in the statistical analyses, while black crosses denote years with less than 100 cases. (B) Mean number of heavy rainfall events in each month across years. The solid and dashed lines correspond to values estimated with only years with more than 100 locally-acquired dengue cases and all years, respectively.

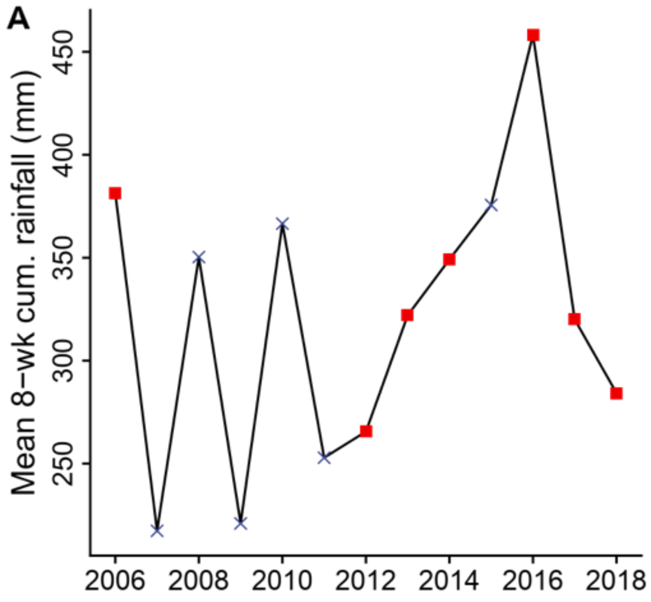

Figure S3. Mean 8-week cumulative rainfall across years or months. (A) Mean 8-week cumulative rainfall in each year. Red squares correspond to years with more than 100 cases, which were included in the statistical analyses, while black crosses denote years with less than 100 cases. (B) Mean 8-week cumulative rainfall in each month. The solid and dashed lines correspond to values estimated with only years with more than 100

locally-acquired dengue cases and all years, respectively.

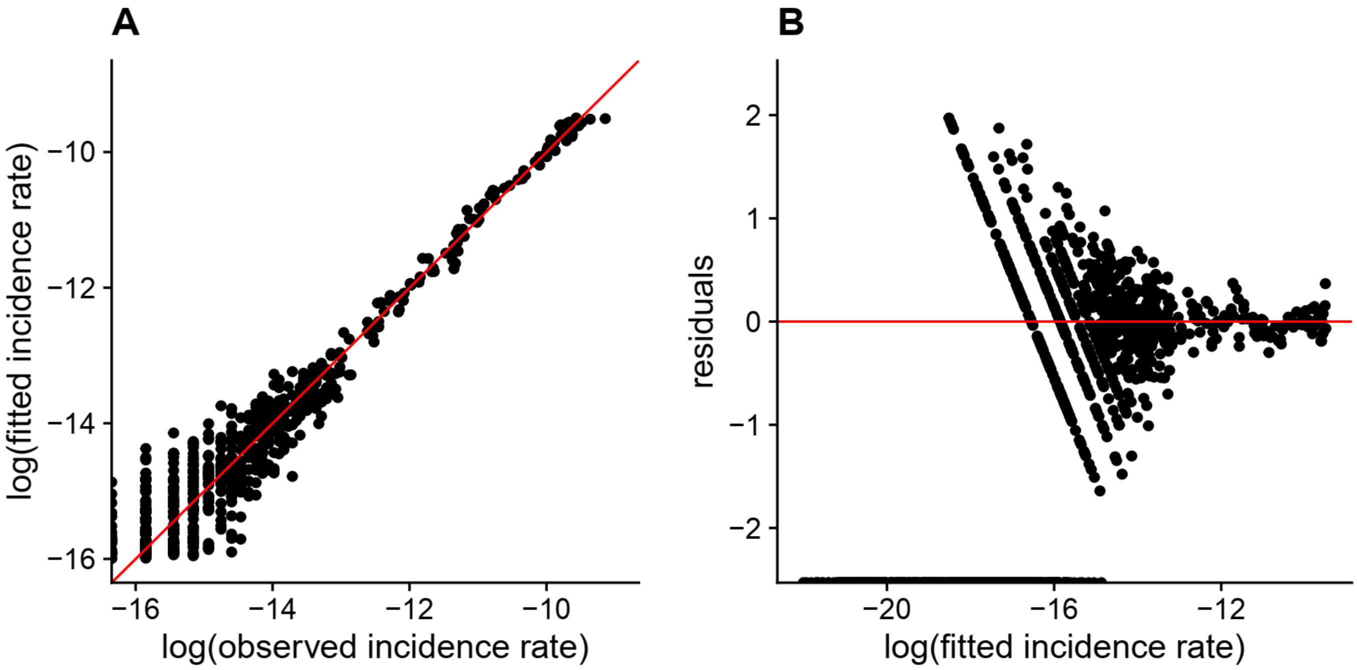

Figure S4. Diagnostic plots of the baseline model.

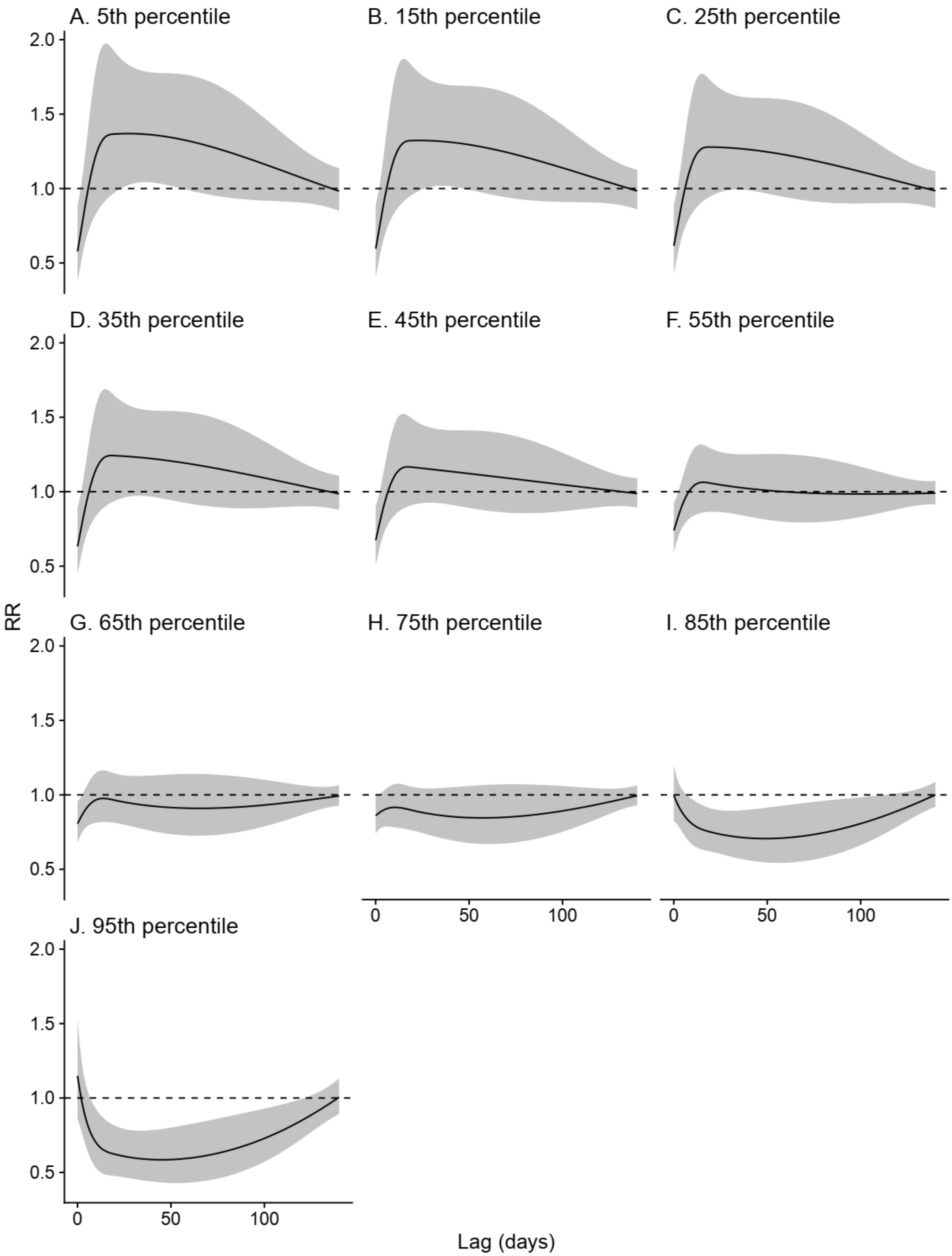

Figure S5. Lagged effects of heavy rainfall events on dengue infection risk by prior water availability scenarios when the centering value for the cumulative rainfall during the preceding 8 weeks (the proxy for prior water availability) changed between its 5th to 95th percentile.

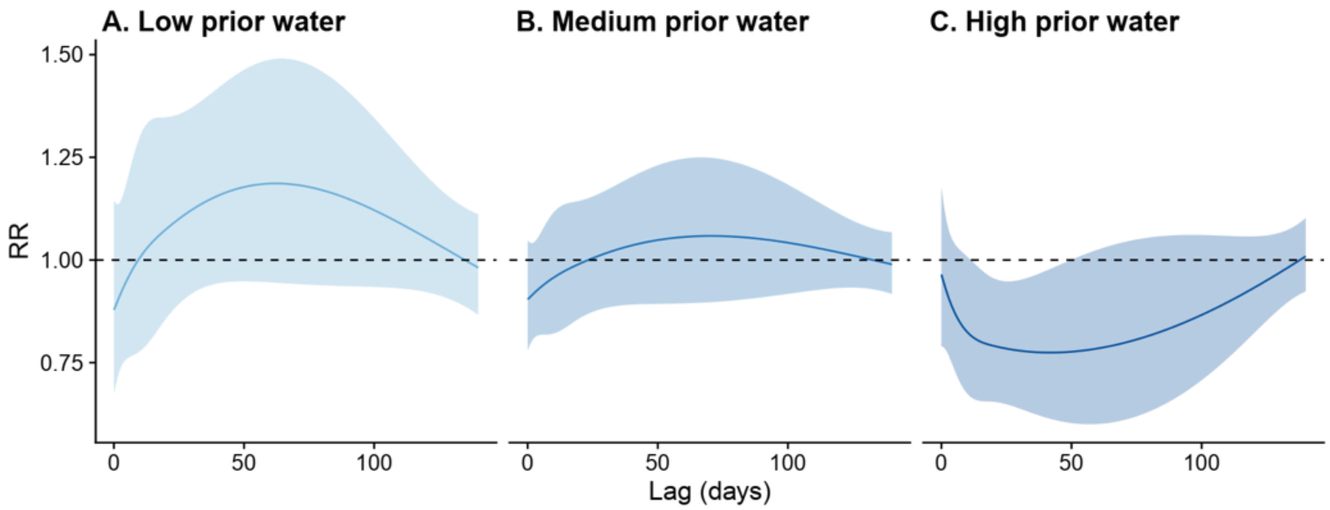

Figure S6. Lagged effects of heavy rainfall events on dengue infection risk by prior water availability scenarios when heavy rainfall was defined as daily precipitation above its 90th percentile value on rainy days in the study period (34.6 mm).

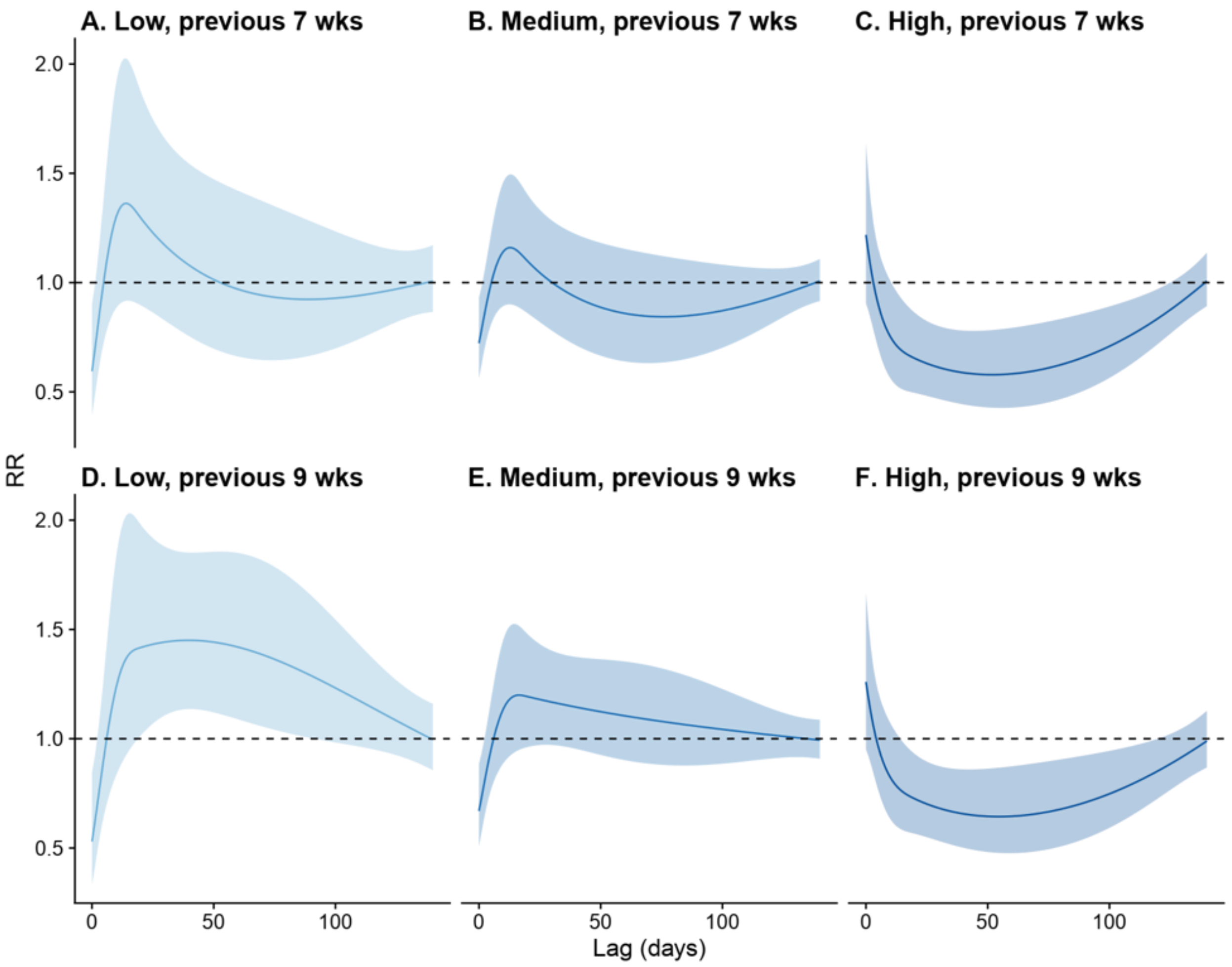

Figure S7. Lagged effects of heavy rainfall events on dengue infection risk by prior water availability scenarios when prior water availability was represented by the cumulative rainfall in the previous 7 (A-C) or 9 (D-F) weeks.

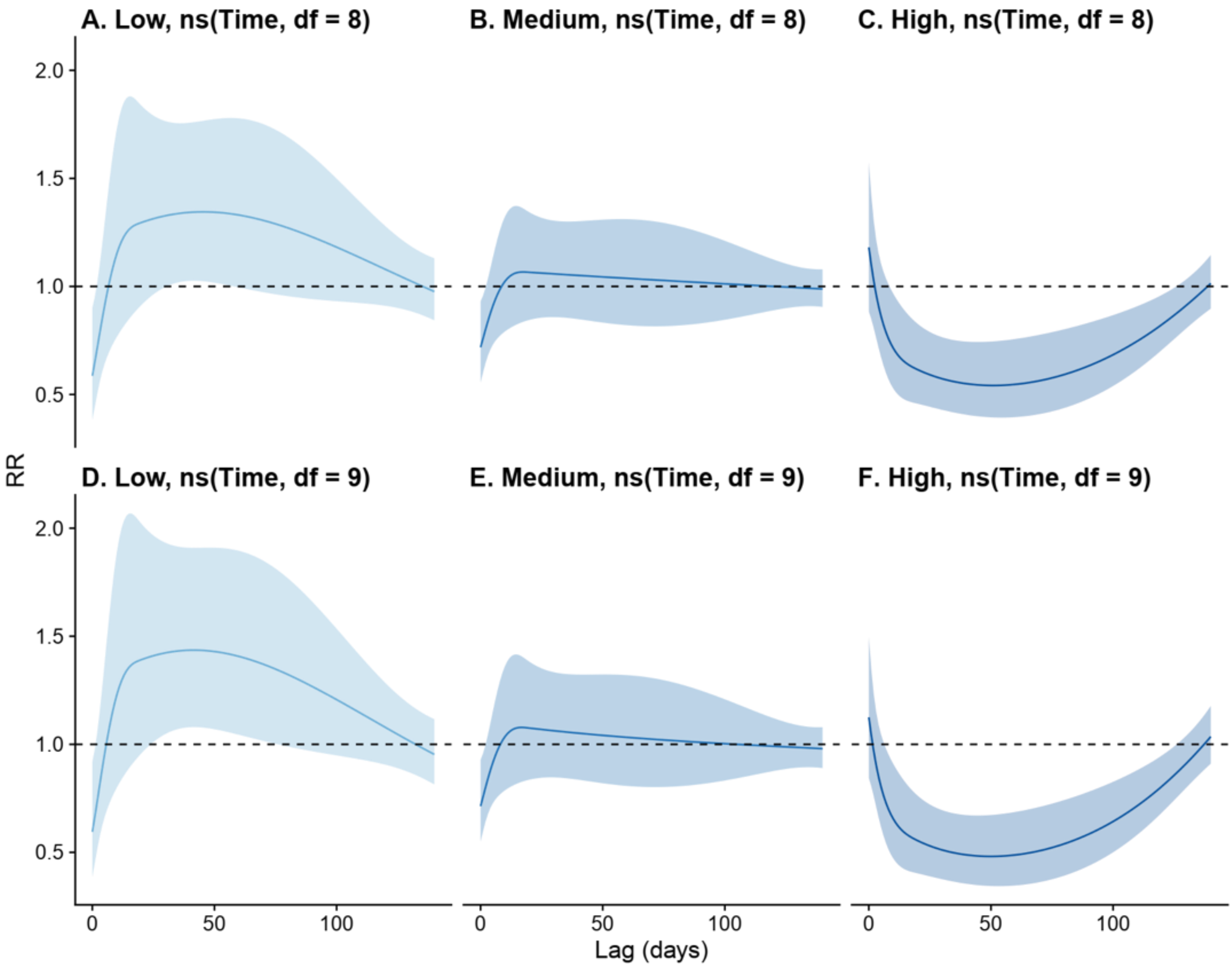

Figure S8. Lagged effects of heavy rainfall events on dengue infection risk by prior water availability scenarios when the degrees of freedom per year used in $ns(Time)$, a smooth function to control for long-term and seasonal trends of potential unmeasured confounders, was set to 8 (A-C) or 9 (D-F).

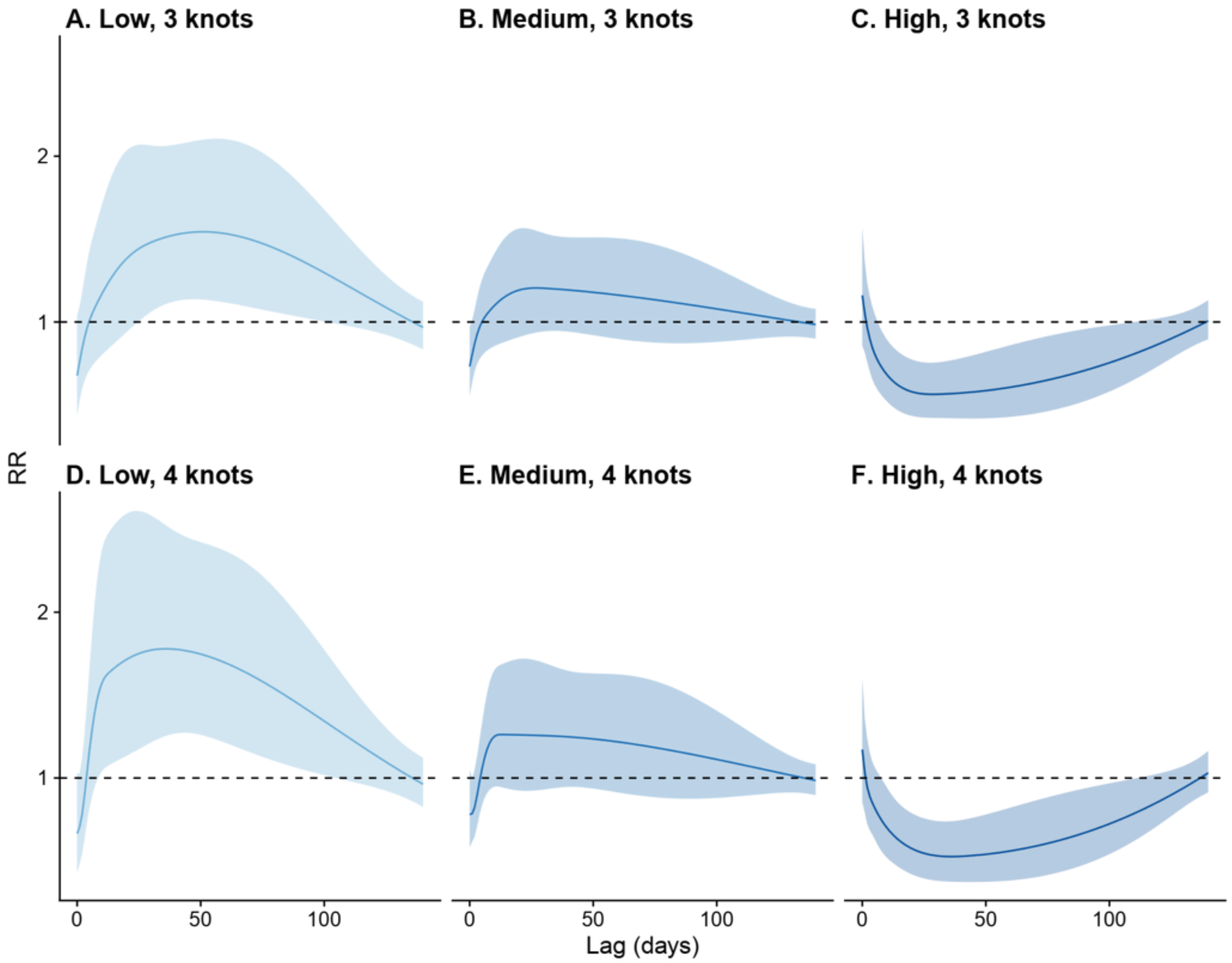

Figure S9. Lagged effects of heavy rainfall events on dengue infection risk by prior water availability scenarios when 3 (A-C) or 4 (D-F) knots was used to specify the lag-response relationship of heavy rainfall events.

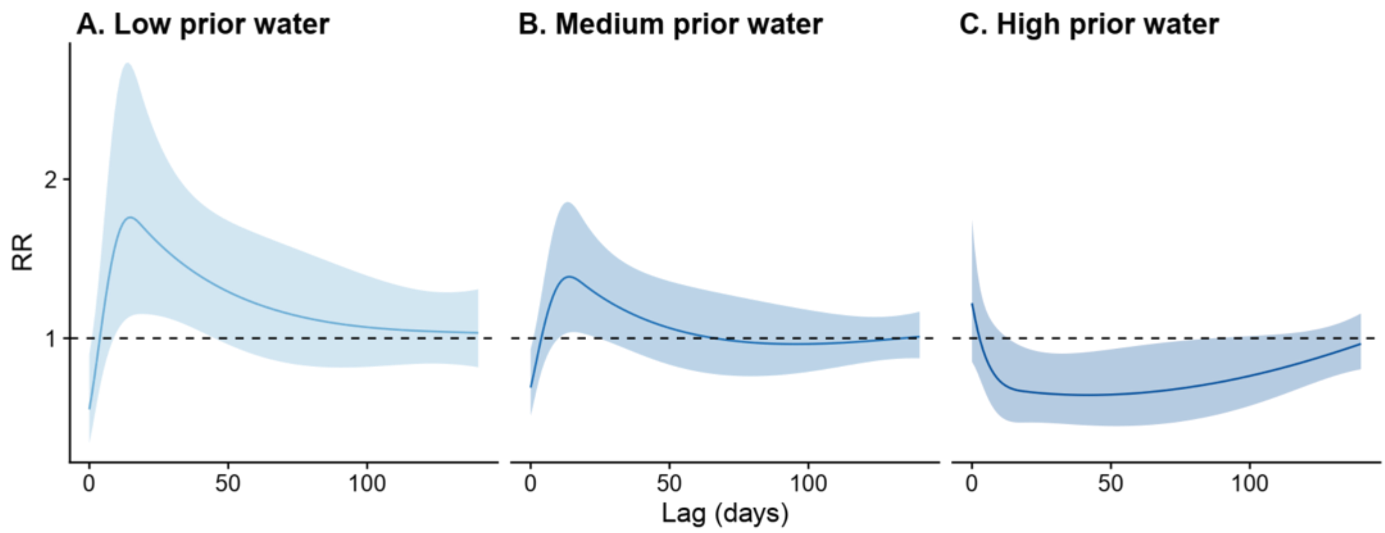

Figure S10. Lagged effects of heavy rainfall events on dengue infection risk by prior water availability scenarios when data from the year 2014 was excluded from the analyses.

Table S1. Summary results of the sensitivity analyses.

Scenarios	Values	Water availability	Lags with significant associations ($p < 0.05$)	Lag day with the strongest association	IRR and its 95% CI on the lag day with the strongest association
Centering value for prior water availability (Fig S4)	15th percentile	N. A.#	24-55	23	1.32, 0.98-1.78
	25th percentile	N. A.	27-47	19	1.28, 0.94-1.73
	35th percentile	N. A.	Not significant	18	1.24, 0.93-1.67
	45th percentile	N. A.	Not significant	17	1.17, 0.90-1.51
	55th percentile	N. A.	Not significant	16	1.06, 0.86-1.31
	65th percentile	N. A.	Not significant	65	0.91, 0.73-1.14
	75th percentile	N. A.	Not significant	57	0.84, 0.67-1.07
	85th percentile	N. A.	7-113	49	0.71, 0.55-0.91
Threshold for heavy rainfall events (Fig S5)	90th percentile	Low	Not significant	62	1.19, 0.95-1.49
		Medium	Not significant	70	1.06, 0.90-1.25
		High	12-49	42	0.77, 0.61-0.98
Number of preceding weeks for estimating prior water availability (Fig S6)	7	Low	Not significant	14	1.36, 0.92-2.03
		Medium	Not significant	13	1.16, 0.90-1.50
		High	11-124	52	0.58, 0.43-0.78
	9	Low	19-91	40	1.45, 1.14-1.85
		Medium	Not significant	16	1.20, 0.95-1.52
		High	15-119	55	0.64, 0.48-0.87
Degrees of freedom per year (Fig S7)	8	Low	31-58	45	1.34, 1.02-1.77
		Medium	Not significant	17	1.07, 0.83-1.36
		High	9-125	51	0.54, 0.39-0.75
	9	Low	24-75	41	1.44, 1.08-1.91
		Medium	Not significant	17	1.08, 0.83-1.41
		High	7-126	50	0.48, 0.34-0.67
Number of knots for the lag-response relationship of heavy rainfall (Fig S8)	3	Low	25-101	51	1.54, 1.13-2.10
		Medium	Not significant	27	1.21, 0.94-1.55
		High	7-110	28	0.56, 0.42-0.75
	4	Low	9-104	36	1.78, 1.25-2.52

	Medium	Not significant	13	1.26, 0.94-1.69
	High	8-111	36	0.53, 0.37-0.74
Remove data from the year 2014 (Fig S9)	Low	9-45	15	1.76, 1.14-2.72
	Medium	10-24	14	1.39, 1.04-1.86
	High	13-86	42	

[#] Not applicable since the centering value for prior water availability was changed between the 15th and 85th percentile values of the cumulative rainfall in the preceding 8 weeks, rather than fixed at its 5th (*low* prior water availability), 50th (*medium* prior water availability), and 95th (*high* prior water availability) percentile values.
